# Effectiveness of an adapted diving mask (Owner mask) for non-invasive ventilation in the COVID-19 pandemic scenario: study protocol for a randomized clinical trial

**DOI:** 10.1186/s13063-022-06133-y

**Published:** 2022-03-18

**Authors:** Dulciane Nunes Paiva, Litiele Evelin Wagner, Sônia Elvira dos Santos Marinho, Carlos Fernando Drumond Dornelles, Juliana Fernandes de Souza Barbosa, Patrícia Érika de Melo Marinho

**Affiliations:** 1 Post-Graduate Program in Health Promotion, Universidade de Santa Cruz do Sul, Santa Cruz do Sul, RS Brazil; 2 Multiprofessional Residency Health Program, Hospital Santa Cruz, Santa Cruz do Sul, RS Brazil; 3 Post-Graduate Program in Physical Therapy, Universidade Federal de Pernambuco, Recife, PE Brazil; 4 Hospital Santa Cruz, Santa Cruz do Sul, RS Brazil

**Keywords:** COVID-19, Clinical trial, Respiratory failure, Non-invasive ventilation, Innovation

## Abstract

**Background:**

Non-invasive ventilation (NIV) is indicated to avoid orotracheal intubation (OTI) to reduce hospital stay and mortality. Patients infected by SARS-CoV2 can progress to respiratory failure (RF); however, in the initial phase, they can be submitted to oxygen therapy and NIV. Such resources can produce aerosol and can cause a high risk of contagion to health professionals. Safe NIV strategies are sought, and therefore, the authors adapted diving masks to be used as NIV masks (called an Owner mask).

**Objective:**

To assess the Owner mask safety and effectiveness regarding conventional orofacial mask for patients in respiratory failure with and without confirmation or suspicion of COVID-19.

**Methods:**

A Brazilian multicentric study to assess patients admitted to the intensive care unit regarding their clinical, sociodemographic and anthropometric data. The primary outcome will be the rate of tracheal intubation, and secondary outcomes will include in-hospital mortality, the difference in PaO_2_/FiO_2_ ratio and PaCO_2_ levels, time in the intensive care unit and hospitalization time, adverse effects, degree of comfort and level of satisfaction of the mask use, success rate of NIV (not progressing to OTI), and behavior of the ventilatory variables obtained in NIV with an Owner mask and with a conventional face mask. Patients with COVID-19 and clinical signs indicative of RF will be submitted to NIV with an Owner mask [NIV Owner COVID Group (*n* = 63)] or with a conventional orofacial mask [NIV orofacial COVID Group (*n* = 63)], and those patients in RF due to causes not related to COVID-19 will be allocated into the NIV Owner Non-COVID Group (*n* = 97) or to the NIV Orofacial Non-COVID Group (*n* = 97) in a randomized way, which will total 383 patients, admitting 20% for loss to follow-up.

**Discussion:**

This is the first randomized and controlled trial during the COVID-19 pandemic about the safety and effectiveness of the Owner mask compared to the conventional orofacial mask. Experimental studies have shown that the Owner mask enables adequate sealing on the patient’s face and the present study is relevant as it aims to minimize the aerosolization of the virus in the environment and improve the safety of health professionals.

**Trial registration:**

Brazilian Registry of Clinical Trials (ReBEC): RBR – 7xmbgsz. Registered on 15 April 2021.

## Administrative information

Note: the numbers in curly brackets in this protocol refer to SPIRIT checklist item numbers. The order of the items has been modified to group similar items (see http://www.equator-network.org/reporting-guidelines/spirit-2013-statement-defining-standard-protocol-items-for-clinical-trials/).

**Table:** 

Title {1}	**EFFECTIVENESS OF AN ADAPTED DIVING MASK (OWNER MASK) FOR NON-INVASIVE VENTILATION IN THE SARS-COV-2 PANDEMIC SCENARIO: A RANDOMIZED CLINICAL TRIAL**
Trial registration {2a and 2b}.	RBR – 7xmbgsz Facial Mask Ventilation IN THE Sars-CoV-2 pandemic scenario
Protocol version {3}	Version 1.0 05 June 2021
Funding {4}	Onwtec Soluções em Engenharia Company – Improvement of diving masks Decathlon Company – Provision of adapted diving masks Universidade de Santa Cruz do Sul – Technological support
Author details {5a}	Departamento de Ciências da Saúde. Santa Cruz do Sul – RS, Brasil. Universidade de Santa Cruz do Sul. Departamento de Ciências da Vida/Programa de Pós-Graduação Mestrado e Doutorado em Promoção da Saúde, Santa Cruz do Sul – RS, Brasil. Universidade de Santa Cruz do Sul. Departamento de Gestão de Negócios e Comunicação. Santa Cruz do Sul – RS, Brasil. Hospital Regional do Agreste. Caruaru – PE, Brasil. Empresa Owntec Soluções em Engenharia. Santa Cruz do Sul – RS, Brasil. Hospital Santa Cruz. Universidade de Santa Cruz do Sul. Santa Cruz do Sul – RS, Brasil.
Name and contact information for the trial sponsor {5b}	Dulciane Nunes Paiva – (55) 51 – 99666-7963 dulciane@unisc.br
Role of sponsor {5c}	**Patrícia Érika de Melo Marinho -** Management, analysis, and interpretation of data; writing of the report; and the decision to submit the report for publication. The author will have final authority over any of these activities. **Litiele Evelin Wagner -** Collection, management, analysis, and interpretation of data; writing of the report. **Juliana Fernandes de Souza Barbosa -** Management, analysis, and interpretation of data; writing of the report; and the decision to submit the report for publication. The author will have final authority over any of these activities. **Sonia Elvira dos Santos Marinho -** Collection, management, analysis, and interpretation of data; writing of the report. **Carlos Fernando Drumond Dornelles -** Interpretation of data and writing of the report. **Dulciane Nunes Paiva -** Management, analysis, and interpretation of data; writing of the report; and the decision to submit the report for publication, including whether they will have ultimate authority. The author will have final authority over any of these activities.

## Introduction

### Background and rationale {6a}

After the emergence of a new coronavirus called SARS-CoV-2, the COVID-19 disease was initially characterized by fever, sore throat, cough and dyspnea, and mainly by manifestations of the respiratory system [1]. Other manifestations, including neurological and micro-hemodynamic findings, were added to its clinical spectrum during the course of the COVID-19 pandemic [2], with 81% of patients having mild symptoms, 14% being severe, and 5% being very severe [3, 4].

Non-invasive ventilation (NIV) traditionally has the indication of avoiding orotracheal intubation (OTI), reducing hospital stay and mortality, being an important resource in the intensive care repertoire [5]. Westhoff et al. [6] showed that NIV was associated with elevated mortality when the ratio of the partial pressure of oxygen in arterial blood (PaO_2_) to the inspired oxygen fraction (FiO_2_) (PaO_2_/FIO_2_) was < 150 mmHg [7], also demonstrating that delayed intubation is prognostically unfavorable.

It should be noted that the recommendations for NIV for patients with COVID-19 are still under discussion, but what has been shown so far is that there is generally a good response to oxygen therapy and NIV in COVID-19 due to low VA/Q ratio (Ltype) [8], with low intrapulmonary shunt with consequent severe oxygenation disorder as in the traditional acute respiratory distress syndrome to accurately indicate OTI.

It is recommended to institute NIV in addition to oxygen supplementation when there is worsening of respiratory discomfort, while OTI and the institution of mechanical ventilation becomes a priority in case of failure in this therapy. It is estimated that 30.8% of people infected with SARS-CoV2 remain asymptomatic, and 15 to 20% among those who are symptomatic require hospitalization, of which 5% progress to a critical condition—a condition which requires more complex interventions [3, 4].

Although the significant escape of infectious aerosols is a risk in NIV, it is recommended that aerosolization is minimized by the use of an appropriate interface and the use of filters such as the high-efficiency particulate arrestance (HEPA) in the expiratory branch [9, 10]. It is additionally recommended that all non-invasive treatments should be monitored closely due to the risk of rapid clinical deterioration [6]. According to Landry et al. [11], mask leaking from positive pressure systems may be a major source of environmental contamination and nosocomial spread of infectious respiratory diseases and that improvised diving masks show greater leaking at higher flow rates compared to standard continuous positive airway pressure (CPAP) masks at pressures greater than 10–12 cmH_2_O. Therefore, NIV should be used with CPAP pressure levels of up to 12 cmH_2_O, with the aim of minimizing aerosolization, maintaining peripheral oxygen saturation (SpO_2_) between 93 and ≤ 96%, inspired fraction of oxygen (FiO_2_) ≤ 50% and respiratory rate (RR) < 24 bpm, which should be evaluated within 1 h [11, 12].

Predicting the lack of materials to meet the needs of these patients in the intensive care units (ICU), the “Mergulhadores do Bem” group from the Universidade de Santa Cruz do Sul—RS/Brazil, inspired by the idea implemented in Italy and used in hospitals (https://www.isinnova.it/easy-covid19/), adapted masks used for diving (snorkeling) to be used as NIV masks, called the Owner mask, which can be adapted to traditional mechanical ventilators and portable non-invasive ones (http: //www.mergulhadoresdobem.com.br/).

Thus, in view of the urgent need to develop measures to assist in ventilatory care for patients with SARS-CoV-2 infection, the Owner mask was designed to prioritize protection and assist in respiratory function, allowing an adequate seal on the patient’s face and minimizing the virus aerosolization in the environment. Considering that patients with COVID-19 can benefit from early non-invasive ventilatory strategy and that the interface for NIV needs to avoid aerosolization for the environment to reverse/minimize the acute respiratory failure (RF), the present study aims to assess the safety and efficacy of the Owner mask in relation to the conventional orofacial mask for performing NIV.

### Objectives {7}

The primary objective of the study is to determine whether the use of Owner mask is effective in reducing the risk of OTI compared to conventional orofacial mask for patients with and without COVID-19 requiring NIV and the secondary objectives were to determine safety and effectiveness by comparing physiological measurements, mortality, length of stay measures, adverse effects, and patient satisfaction.

### Trial design {8}

This is a three-phase, parallel group, randomized, non-blinded, controlled, multicentric study guided by outcome. The allocation ratio is 1:1 in favor of the Owner mask to maximize learning about the experimental treatment, while also allowing a wider pool of patients to have access to the experimental treatment in order to support recruitment.

## Methods: participants, interventions, and outcomes

### Study setting {9}

The setting involved the following Brazilian hospitals: Hospital Santa Cruz (HSC) of Universidade de Santa Cruz do Sul, Hospital Geral Otávio de Freitas (HGOF), and Hospital Regional do Agreste—Universidade Federal de Pernambuco.

### Eligibility criteria {10}

The inclusion criteria are as follows: age ≥ 18 years at time of signing of informed consent form, patients with or without SARS-CoV-2 suspicious or confirmation of both genders, clinical indication to implement NIV, chronic obstructive pulmonary disease exacerbation with hypercapnic acute respiratory failure, congestive heart failure (NYHA 4), hospitalized with SpO_2_ ≤ 94% on room air or PaO_2_/FiO_2_ ≤ 300 mmHg, willingness of study participant to accept randomization to any assigned treatment arm, and must agree to not enroll in any other study of an antiviral agent prior to completing the 28-day follow-up.

### Exclusion criteria

The exclusion criteria are as follows: comatose patients (Glasgow Coma Scale < 8) or unable to protect the airways; patients who refuse to receive NIV; presence of facial anomalies, facial trauma or facial burn; severe hematemesis or massive hemoptysis; hemodynamically unstable patients (systolic blood pressure (SBP) < 80 mmHg) or receiving vasopressors/inotropes: ongoing angina/acute myocardial infarction or recently developed arrhythmia with hemodynamic impact; patients who underwent esophageal or recent upper respiratory tract surgery (≤ 2 weeks); cardiorespiratory arrest; and patients or legal guardian who do not sign the informed consent form.

### Who will take informed consent? {26a}

Informed consent will be obtained from eligible patients or their substitute decision-makers (for patients lacking decision-making capacity) by study physicians and physiotherapists or other trial staff with delegated responsibility.

### Additional consent provisions for collection and use of participant data and biological specimens {26b}

Not applicable.

### Interventions

#### Explanation for the choice of comparators {6b}

The active arm is the use of the Owner mask to implement NIV, in addition to routine supportive care. The Owner mask (Owntec Soluções em Engenharia, Santa Cruz do Sul, Brazil) was chosen for evaluation because there is safety data from the pilot study which proved reduced air leakage due to its better seal on the patient’s face. The control arm is composed of patients who will undergo NIV using the traditionally used orofacial interface.

#### Intervention description {11a}

**Fig. 1:**
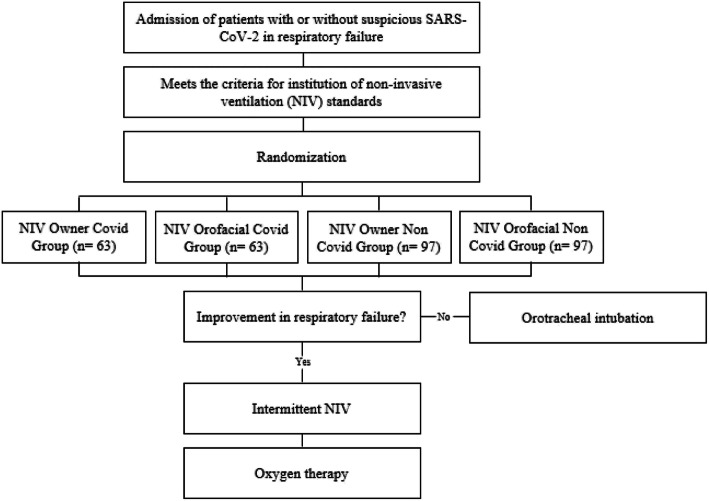
Flowchart of the study

Patients admitted to the emergency or ICU will be screened and recruited using a convenience sample (Fig. 1). Those with suspicion or confirmation of COVID-19, with clinical signs indicative of respiratory insufficiency, will be submitted to NIV with Owner mask (Group NIV Owner COVID) or with conventional orofacial mask (Group NIV orofacial COVID), and those patients in RF due to causes not related to COVID-19 will be allocated into the NIV Owner Group or to the orofacial NIV Group in a randomized way.

NIV will be instituted in CPAP or bi-level positive airway pressure (BiPAP) mode with expiratory positive airway pressure (EPAP) and inspiratory positive airway pressure (IPAP) levels of up to 10–12 cmH_2_O. NIV will be used for 30 min to 1 h for patients with COVID-19, with gas analysis before and after this period. Sessions in non-COVID-19 patients can last up to 2 h, and NIV can be used intermittently, as long as there is no clinical worsening or signs of failure [13, 14].

- CPAP: hypoxemic respiratory failure, with positive expiratory end pressure (PEEP) between 5 and 12 cmH_2_O and FiO_2_ minimum to maintain SpO_2_ ≥ 94%
- BIPAP: hypercapnic respiratory failure, start with IPAP necessary to maintain SpO_2_ > 94% and with maximum levels up to 10–12 cmH_2_O. Tidal volume up to a maximum of 9 ml/Kg/predicted weight, EPAP (PEEP) between 6 and 8 cmH_2_O and FiO_2_ minimum enough for SpO_2_ ≥ 94%

NIV will be performed by a conventional mechanical ventilator, preferably by SERVO-S (Maquet Critical Care, São Paulo, Brazil), or by the one available at each research center. The criteria for its interruption will be those of clinical worsening (determined by the medical team) such as the occurrence of hemodynamic instability, depression of the cognitive state, psychomotor agitation, lack of airway defense, lack of adaptation to the interface, and severe bronchospasm [15]. Success or reduction in the severity of RF will be considered by the oxygenation index through the PaO_2_/FiO_2_ > 200, reduction in RR < 30 bpm and SpO_2_ ≥ 94% [12, 16].

1. NIV Owner group (experimental group)
  Patients with suspected or confirmed COVID-19 undergoing NIV with an Owner mask
  Patients with RF due to causes unrelated to COVID-19 submitted to NIV with Owner mask
2. NIV orofacial group (control group)
  Patients with suspected or confirmed COVID-19 undergoing NIV with conventional orofacial mask
  Patients with RF due to causes unrelated to COVID-19 submitted to NIV with conventional orofacial mask

#### Criteria for discontinuing or modifying allocated interventions {11b}

Adverse events of the NIV interface can be due to both the adaptation of the orofacial mask or the Owner mask, and treatment will be stopped in the case of any events occurring which are considered serious or intolerable in the judgment of the investigators and the data monitoring committee (DSMB) and under medical consent.

Interruption of the experimental treatment will occur by:

- Air leakage around the mask with air leak detected in the mechanical ventilator > 24 l/min
- Patient-mechanical ventilator asynchrony due to the occurrence of leakage around the mask
- Pressure of fixing the mask against the excessive face that may cause the risk of skin ulceration
- Traumatic skin injury due to direct contact with the mask
- Signs of corneal dryness
- Discomfort related to the use of the mask
- Phobia of staying in a closed environment, with signs of intolerance to the adaptation of the mask
- Psychomotor agitation after adaptation of the mask
- Level of 10 mmHg CO_2_ increase from baseline

Individuals who declare they no longer wish to participate at any time or who present criteria for interrupting NIV will be discontinued from the study, according to the recommendations of the *Associação de Medicina Intensiva Brasileira* (*AMIB*) [15].

#### Strategies to improve adherence to interventions {11c}

This item is not applicable, since NIV is a resource which has its clinical indication established [15].

#### Relevant concomitant care permitted or prohibited during the trial {11d}

All concomitant care and interventions are permitted other than concomitant receipt of any other experimental treatment. Patients will use the drug therapy indicated by the attending physician. The NIV will be implemented by using a diving mask adapted into a mechanical ventilator, a HEPA filter in the expiratory branch, and a heat and moisture exchanger (HME) filter in the trachea Y, with it being performed twice a day for 60 min., with positive pressure levels of up to 10–12 cmH_2_O whenever there is a clinical indication to do so [12].

#### Provisions for post-trial care {30}

No special post-research regime is anticipated.

### Outcomes {12}

#### Primary outcome measure

OTI rate in patients with or without suspicion or confirmation of SARS-CoV-2 infection submitted to NIV with Owner mask or conventional orofacial mask.

#### Secondary outcome measure

- In-hospital mortality [period: up to 30 days]
- Differences in the PaO_2_/FiO_2_ ratio regarding the baseline values (before NIV) [period: 48 h]
- Improvement in the PaO_2_/FiO_2_ ratio after beginning NIV [period: 1 h after NIV]
- PaCO_2_ levels [period: 1 to 12 h after NIV]
- Blood pressure (BP) and hypotension incidence (SBP < 80 mmHg or mean arterial pressure < 60 mmHg) [period: up to 2 h after NIV]
- Time between beginning NIV and its end
- ICU time [time period: up to 30 days]
- Length of hospital stay of patients who used NIV
- Adverse events of the mask [period: obtained at pre-hospital discharge]
- Comfort of the patient with the mask (visual analog scale—VAS) [period: obtained at pre-hospital discharge]
- Satisfaction level (PGIC) after using NIV [period: obtained at pre-hospital discharge]
- NIV success rate with masks [deadline: 1 h since the beginning of NIV]
- Tidal volume (Vt), minute volume (MV), peak pressure (Ppeak), respiratory rate (RR), FiO_2_, inspiratory time (Ti), expiratory time (Te), air leak, inspiratory minute volume (VMi), and expiratory minute volume (VMe) obtained in NIV [period: 1 h after NIV]

#### Participant timeline {13}

**Fig. 2:**
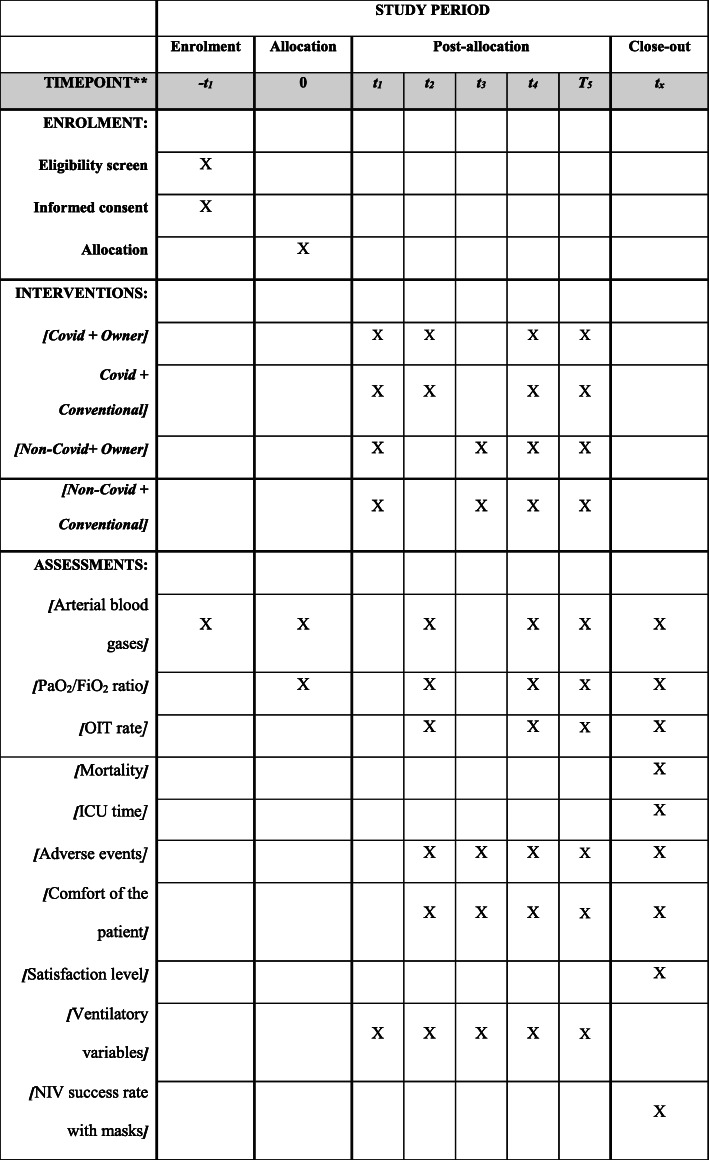
Study timelines of participants/interventions. * t_1_, start; t_2_, 1 h after; t_3_, 2 h after; t_4_, 24 h after; t_5_, 48 h after; t_x_, after the outcome; PaO_2_/FiO_2_, ratio of the partial pressure of oxygen in arterial blood (PaO_2_) to the inspired oxygen fraction (FiO_2_); OIT, orotraqueal intubation; ICU, intensive care unit; NIV, non-invasive ventilation

The participant timeline is shown in Fig. 2.

#### Sample size {14}

The calculated sample size is based on an OIT rate of 30.2% for patients with COVID-19 who require mechanical ventilation [17]. Thus, we estimate that a total of 126 patients (63 in each group) would provide 80% of power to detect an absolute reduction of 20% of the primary result [18], with the adoption of a two-tailed model and a significance level of 0.05. Assuming a loss of follow-up of 20%, 151 patients will be recruited.

Assuming an intubation rate of 61% for patients with acute respiratory syndrome requiring mechanical ventilation [19], we calculated that a total of 194 (97 in each group) patients would provide 80% power to detect an absolute reduction 20% of the primary result [18], with the adoption of a two-tailed model and a significance level of 0.05. Assuming a loss of follow-up of 20%, 232 patients will be recruited. The sample size calculations were performed using the G*Power software (Version 3.1.9.6) [20]. A total of 383 patients will participate in the study, 151 in the group of patients with suspicion or confirmation SARS-CoV-2, and 232 for those with respiratory syndrome who require non-invasive ventilation.

#### Recruitment {15}

Patients will be recruited from the designated hospitals for patients with and without COVID-19 with indication to receive NIV. The sample will be recruited non-probabilistically and by convenience, in which patients who meet the criteria for NIV institution will be selected according to the AMIB [12]. All hospitals involved in the study are a reference for the care of patients with or without COVID-19 and who need ventilatory assistance.

### Assignment of interventions: allocation

#### Sequence generation {16a}

The choice of the traditional orofacial mask or the Owner mask will be carried out by randomization performed using a random block table (10 patients per block) generated by the randomization.com program for patient allocation.

#### Concealment mechanism {16b}

Eligible patients will be allocated to receive NIV with Owner mask or conventional orofacial mask in individually numbered packs according to the sequential order provided by a person who will not be engaged in the study. Each treatment group will be coded and the allocation will be transferred to a series of opaque numbered envelopes for the patients selected for the study. The envelopes will be sent to the researcher responsible for the treatment phase.

#### Implementation {16c}

Patients will receive NIV through the Owner mask or the conventional orofacial mask according to the sequence of the previous randomization sent to each hospital. The choice of the traditional orofacial mask or the Owner mask will occur by randomization performed by a random table generated by a computer program that will determine the patient allocation group and the treatment allocation will be determined by the treating team opening the next sealed envelope. Both groups will be allocated to receive NIV by adapted mask or conventional mask with COVID-19 confirmed, or with other respiratory diseases. The random table will be the same for both groups of patients (with COVID-19 and/or another respiratory disease).

### Assignment of interventions: blinding

#### Who will be blinded {17a}

Only the data analyst will be blinded.

#### Procedure for unblinding if needed {17b}

Not applicable.

### Data collection and management

#### Plans for assessment and collection of outcomes {18a}

The data of each research subject (including discontinued subjects) will be registered in the database quickly, completely, and accurately through an electronic form made available to each researcher in their respective hospital and mandatory to be completed and checked by the researchers investigated by the study in each hospital institution. The data administrator will review and modify the data according to the check of the researchers where the research is being carried out.

Analysis of clinical, sociodemographic data, age, sex, and body mass index (BMI) will be performed. An analog pain assessment will subsequently be performed using the VAS [21] and the perception of effort using the Modified Borg Scale. Clinical severity will be quantified using the Simplified Acute Physiological Score III (SAPS III) based on data obtained from the patient’s medical record. The presence of comorbidities, smoking, symptoms, confirmation of SARS-CoV-2 infection, origin of the patient, history of exposure, and complications that may occur during hospitalization will be recorded. The OTI rate will be obtained in patients who have undergone NIV, as well as in-hospital mortality within a period of up to 30 days.

#### Anthropometric data

Body mass (Kg), height (meters) and subsequently the BMI will be obtained through the ratio between body mass and height squared (kg/m^2^) [22]. The ideal body mass (Kg) will be obtained through the estimate given by *Diretrizes Brasileiras de Ventilação Mecânica da AMIB* [15]. The height will be measured by means of an inextensible and inelastic anthropometric tape of 1.5 m and an interval of 0.01 m. The semi-wingspan will be obtained by measuring the distance between the sternum and the tip of the middle finger of one hand (hemi wingspan) [23, 24]. In an effort to minimize the leak and the factors that can predispose to a mask leak, the beard/facial hair will be removed before the institution of the NIV.

#### Vital signs and respiratory effort

SpO_2_ will be measured using pulse oximetry (DX2010, Dixtal, Manaus, AM, Brazil), and BP and heart rate (HR) will be assessed through digital monitoring (DX2010, Dixtal, Manaus, AM, Brazil). Hypotension will be considered when SBP < 80 mmHg or mean arterial pressure < 60 mmHg) within 24 h after NIV. RR will be obtained by a plethysmographic signal provided by the VM monitor (SERVO-S, Maquet Critical Care, São Paulo, SP, Brazil). The respiratory effort will be qualified by inspection when the patient develops tachypnea, use of accessory muscles, flapping of the nose, change in breathing pattern, shallow breathing, and abnormal movements of rib cage [25]. Such measures will be performed before and after 1 h of NIV institution.

#### Ventilatory parameters

The following ventilatory variables will be monitored in the mechanical ventilation: Vt, MV, Ppeak, RR, FiO_2_, Ti, Te, air leak, VMi, and VMe. Such variables will be obtained before and after the application of NIV. Such measures should be carried out after 1 h of NIV adaptation.

#### Arterial blood gas analysis

Arterial blood gasses will be obtained according to the routines of each center participating in the research. For research purposes, the gasometric variables will be assessed at the baseline (before NIV) and from 1 to 12 h from the beginning of NIV.

#### PaO_2_/FiO_2_ ratio

The difference in PaO_2_/FiO_2_ ratio regarding the baseline values (before NIV) will be assessed within 48 h, and the improvement of such relationship after the beginning of NIV, within 1 h after NIV.

#### Comfort of the NIV face mask

The comfort provided by facial masks will be assessed before hospital discharge using the VAS, if the patient presents a satisfactory response to NIV with the Owner mask and the conventional orofacial mask and presents a stable clinical condition.

#### Adverse effects of the NIV face mask

The adverse effects of wearing the masks will be assessed before the patient’s discharge from the hospital using a questionnaire consisting of 11 questions regarding: pain in the face region (forehead, nose, cheeks or chin), leaks (around the eyes and around the mouth), dryness (mouth, throat and nose), pressure, irritation, or claustrophobia [26].

#### Global Perception of Change

The Patients’ Global Impression of Change (PGIC) questionnaire will be used to assess the perception of improvement in individuals submitted to NIV use with the Owner mask before the patient’s discharge from hospital [27].

#### Plans to promote participant retention and complete follow-up {18b}

The procedures will be interrupted due to the occurrence of unexpected events, in which the necessary assistance will be provided immediately. It is noteworthy that the data collection will not interfere in the hospital routine of the evaluated patients nor in any medical treatment that the patient is developing. If necessary, the right to immediate, full and free healthcare will be guaranteed.

The patients will not incur any expenses with transportation, food, exams, and materials to be used or expenses of any other nature to participate in this study. Compensation will occur in the event of any damages resulting from the study. Patients will also receive clarification on any questions about the procedures, risks, benefits, and other issues related to the research. The patient may stop participating in the study without prejudice to continuing their treatment. Medical treatment and compensation will be offered if there is damage to the patient’s health caused by this study.

#### Data management {19}

The data will initially be inserted in an Excel spreadsheet with double data entry and will be checked weekly by the researchers responsible for each hospital before being sent to a spreadsheet where all of this data will be computed and will be under the responsibility of the project coordinator. All variables will be categorized and coded for security, where only those responsible for the co-participating institutions will have access to filling, checking, and storing. Researchers will be responsible for evaluating and collecting results from the baseline to the end of the study. All data will be verified again by the research coordinator and the associates responsible for each hospital unit involved, as well as by the DSMB.

#### Confidentiality {27}

The data of the evaluated patients will be collected and identified by a single identification of the study subject, preserving anonymity, as well as the samples collected through the study. The record linking the study subject’s identification with the patient’s identification information will be kept confidential at each recruitment location.

#### Plans for collection, laboratory evaluation, and storage of biological specimens for genetic or molecular analysis in this trial/future use {33}

These plans are not yet in place.

### Statistical methods

#### Statistical methods for primary and secondary outcomes {20a}

The SPSS software program (version 25.0, IBM, Armonk, NY, USA) will be used for data analysis using the Generalized Estimation Equations (GEE) model. The normality of the data will be assessed using the Kolmogorov-Smirnov test. Identity function, unstructured work correlation matrix, and covariance matrix of the robust estimator will be used using the Bonferroni post hoc test when significant. The chi-squared test will be used to compare the proportions of categorical variables studied in the groups and the Mann-Whitney *U* test to compare the distributions of continuous variables between groups (*p* < 0.05). Analysis by intention-to-treat will be performed to include patients who did not allow analysis of outcomes.

The effect size will be estimated according to Cohen [28], who suggested a cutoff value greater than or equal to 0.8 representing a large effect size, between 0.8 and 0.2 as a medium effect size, and values below 0.2 as a small effect size. The same will be calculated using the variation obtained before and after the intervention, as well as for the post-intervention moment between the groups analyzed. The effect size will be calculated using Dr. Lee A. Becker’s Effect Size Calculator [http://www.uccs.edu/lbecker/].

#### Interim analyses {21b}

DSMB recommendations will be notified directly to the research coordinator who will communicate to all parties involved in the research. The DSMB will carry out statistical analysis at a frequency to be established by the committee to detect the need to discontinue the study because of the primary outcome or due to the risk of occurrence of serious adverse outcomes, as well as ongoing assessment of the safety and relevance of the study design. The primary outcome of the study will be considered: OTI rate in patients with or without suspicion or confirmation of SARS-CoV-2 infection submitted to NIV with an Owner mask or with a conventional orofacial mask.

#### Methods for additional analyses (e.g., subgroup analyses) {20b}

Statistical analysis will be performed using the generalized estimating equations (GEE) model. The chi-squared test will be used to compare the proportions of categorical variables studied in the groups and the Mann-Whitney *U* test to compare the distributions of continuous variables between groups (*p* < 0.05). The effect size will be estimated according to Cohen [28]. It will be calculated using the variation obtained before and after the intervention, as well as for the post-intervention moment between the analyzed groups.

#### Methods in analysis to handle protocol non-adherence and any statistical methods to handle missing data {20c}

The primary analysis will use the intention-to-treat principle and a per protocol analysis will be undertaken to assess the robustness of the findings.

#### Plans to give access to the full protocol, participant level-data and statistical code {31c}

These plans are not yet in place.

### Oversight and monitoring

#### Composition of the coordinating center and trial steering committee {5d}

##### Coordinating center

The study is led by the Hospital Santa Cruz, Hospital Geral Otávio de Freitas and Hospital Regional do Agreste.

### Trial steering committee

#### The trial steering committee consists of the following members

- Dulciane Nunes Paiva—Departamento de Ciências da Saúde. Programa de Pós-Graduação em Promoção da Saúde da Universidade de Santa Cruz do Sul, RS, Brasil. ORCID: https://orcid.org/0000-0001-5629-3285. E-mail: dulciane@unisc.br
- Patrícia Érika de Melo Marinho—Departamento de Fisioterapia. Programa de Pós-Graduação em Fisioterapia da Universidade Federal de Pernambuco, PE, Brasil. ORCID: https://orcid.org/0000-0002-3093-7481. E-mail: patmarinho@yahoo.com.br
- Juliana Fernandes de Souza Barbosa—Departamento de Fisioterapia. Programa de Pós-Graduação em Fisioterapia da Universidade Federal de Pernambuco, PE, Brasil. ORCID: https://orcid.org/0000-0002-4555-2808. E-mail: julianaferso@gmail.com

#### Trial operation committee

The trial operation committee is comprised of Prof^a^. Patricia Érika de Melo Marinho of Hospital Geral Otávio de Freitas and Regional do Agreste and Prof^a^. Juliana Fernandes de Souza Barbosa.

#### Trial monitoring

Trial monitoring is performed by Prof^a^. Dulciane Nunes Paiva.

#### Data management team

The data management team is comprised of Prof^a^. Juliana Fernandes de Souza Barbosa, Prof^a^. Patrícia Érika de Melo Marinho, and Prof^a^. Dulciane Nunes Paiva.

#### Clinical research organization

The clinical research organization is comprised of Prof^a^. Juliana Fernandes de Souza Barbosa, Prof^a^. Dulciane Nunes Paiva, Prof^a^. Patricia Érika de Melo Marinho, and Dr. Carlos Fernando Drumond Dornelles.

#### Composition of the data monitoring committee, its role and reporting structure {21a}

The independent DSMB in this study is responsible for reviewing the reports regarding the safety of the study patients and protocol adherence and making recommendations to continue or terminate the study or modify sample size on the basis of the results from the interim analysis. The DSMB members are all independent of the sponsor and have no financial or other conflicts of interest.

The coordinator responsible for the study will send monthly reports containing the clinical efficacy and safety data collected during the study, which will be presented in spreadsheets, reports, and documents and will be forwarded to the members of the DSMB. Patients will be coded by numbers for blind analysis. All DSMB recommendations will be notified directly to the research coordinator who will communicate it to all parties involved in the study. The meetings will be held bimonthly via teleconference and the agenda for each meeting will be based on the discussions and recommendations generated in the previous meetings and on the events that may have occurred between them.

#### Data monitoring committee (DSMB) members

Table 1 lists the DSMB members.

**Table 1:** Data monitoring committee members

Name	Role	Position
Brivaldo Markman Filho	Chair	Professor of Universidade Federal de Pernambuco (UFPE)—Chefe do Serviço de Cardiologia do Hospital de Clínicas da UFPE
Maria Cristina Falcão Raposo	Member	Professor of UFPE—Probability and Statistics Area, with emphasis on Applied Probability and Statistics
Leonardo Gasperini	Member	Intensive care physiotherapist and emergency room at Hospital Nossa Senhora da Conceição (HNSC)

#### Adverse event reporting and harms {22}

Any adverse event which may occur will be registered in the database and will be accessed daily by the research coordinator and by the DSMB president designated to monitor this research. The DSMB will perform statistical analysis at intervals established by the committee to detect the need to interrupt the study due to the primary outcome or due to the risk of serious adverse outcomes, as well as continued assessment of the safety and pertinence of the study design.

#### Frequency and plans for auditing trial conduct {23}

The frequency of statistical analysis by the DSMB will be defined by this committee to ensure the safety and well-being of the research participants and the validity of the results obtained, as well as to monitor the occurrence of risks and benefits and detect evidence of achieving the primary objective of the research.

#### Plans for communicating important protocol amendments to relevant parties (e.g., trial participants, ethical committees) {25}

Changing in the study should generate protocol amendments, which will be submitted for analysis by the Ethics Committee/institutional review board. These changes will only be implemented after approval by the ethical committee, and all protocol amendments will also be sent to the DSMB.

#### Ancillary and post-trial care {30}

No special post-research regime will be anticipated.

#### Dissemination plans {31a}

The results of this study will be communicated to health authorities, health professionals, and the general public, at events and through publications in scientific journals as soon as the results are available.

## Discussion

NIV is the delivery of ventilatory support without the need for an invasive artificial airway. The use of non-invasive positive-pressure ventilation in acute respiratory failure has been steadily increasing for ICU patients [29]. NIV can often eliminate the need for intubation or tracheostomy and preserve normal swallowing, speech, and coughing mechanisms. Device discomfort is one of the reasons for NIV failure (30–40% of the cases) [30].

This randomized, controlled study aims to assess the safety and efficacy of the Owner mask in relation to the conventional orofacial mask for implementing NIV in patients in respiratory failure with and without confirmation or suspicion of COVID-19. This is the first randomized clinical trial on the use of a mask adapted for NIV in patients with COVID-19 that we are aware of. Thus, the prevailing recommendations regarding NIV are based primarily on physicians’ experience and on studies in other categories of patients [6, 31]. However, there are a large number of randomized clinical trials on the use of NIV in conditions other than COVID-19 [32–38].

The crucial outcome parameters in most studies are avoidance of intubation and reducing hospital stay and mortality. NIV is an additional measure at the beginning of the disease process as part of a stepwise approach, at a time when the criteria for intubation are not yet fulfilled, having the power to delay or even prevent the need for intubation [5].

An important epidemiological study showed that NIV was associated with elevated mortality when the PaO_2_/FIO_2_ ratio was < 150 mm Hg [7]. This agrees with the conclusion reached by earlier study, i.e., that delayed intubation is prognostically unfavorable [6]. Therefore, the use of NIV must be strictly controlled.

The Owner mask was experimentally evaluated to ensure proper sealing for the patient’s face. Adaptations were made with the placement of valves produced in stainless steel which connect the mask to the mechanical fan and connections printed in 3D, allowing its adapted use for mechanical ventilation and portable NIV. The Owner mask is a flexible, transparent mask shell with a cushion which adapts well to the wearer’s face. The pliable ring embedded inside the flexible clear shell allows the mask to be bent and adjusted to fit the patient’s face (customized fit), while minimizing leakages.

Considering the severity of patients affected by COVID-19 and who develop acute respiratory failure, implementing a study in which the effectiveness of an alternative mask can provide relief of the respiratory condition with comfort and safety, in addition to reducing the chances of OTI and use of invasive mechanical ventilation, seems to have a positive impact in the times of this pandemic. The aim of this study is to evaluate the safety and effectiveness of the diving mask adapted for NIV, since patient compliance and the mechanical characteristics of the delivery devices are two fundamental variables in the success of NIV during acute respiratory failure. We hypothesize that an improved patient-ventilator interface may improve therapy effectiveness.

## Trial status

Brazilian Registry of Clinical Trials (ReBEC), RBR – 7xmbgsz. The protocol version is number 1.0, dated 15 April 2021. https://ensaiosclinicos.gov.br/rg/RBR-7xmbgsz

The first patient, first visit was on 1 September 2020; the recruitment will be completed on 30 April 2022.

## Abbreviations

AMIB: Associação de Medicina Intensiva Brasileira
BiPAP: Bi-level positive airway pressure
BMI: Body mass index
BP: Blood pressure
Bpm: Breathing per minute
COVID-19: Coronavirus disease 2019
CPAP: Continuous positive airway pressure
cmH_2_O: Centimeter of water
DSMB: Data monitoring committee
EPAP: Expiratory positive airway pressure
FiO_2_: Inspired fraction of oxygen
GEE: Generalized estimation equations
HME: Heat and moisture exchanger
HEPA: High-efficiency particulate arrestance
HGOF: Hospital Geral Otávio de Freitas
HR: Heart rate
HSC: Hospital Santa Cruz
ICF: Informed consent form
ICU: Intensive care units
IPAP: Inspiratory positive airway pressure
MV: Minute volume
NYHA: New York Heart Association
NIV: Non-invasive ventilation
OTI: Orotracheal intubation
PaCO_2_: Partial pressure of carbon dioxide in arterial blood
PaO_2_: Partial pressure of oxygen in arterial blood
PaO_2_/FiO_2_: Ratio of the partial pressure of oxygen in arterial blood (PaO_2_) to the inspired oxygen fraction (FiO_2_)
PEEP: Positive expiratory end pressure
PGIC: Patients’ Global Impression of Change
Ppeak: Peak pressure
RF: Respiratory failure
RR: Respiratory rate
RT-PCR: Reverse transcription polymerase chain reaction
SAPS III: Simplified acute physiological score III
SARS-CoV2: Severe acute respiratory syndrome-related coronavirus 2
SBP: Systolic blood pressure
SpO_2_: Peripheral oxygen saturation
SPSS: Statistical Package for the Social Sciences
VA/Q: Ventilation/perfusion ratio
Vt: Tidal volume
VMe: Expiratory minute volume
VMi: Inspiratory minute volume
VAS: Visual analog scale
Te: Expiratory time
Ti: Inspiratory time
USA: United States of America

## References

[1] Guan WJ, Zhong NS (2020). Clinical characteristics of coronavirus disease 2019 in China. N Engl J Med.

[2] Mao L, Jin H, Wang M, et al. Neurologic manifestations of hospitalized patients with coronavirus disease 2019 in Wuhan, China. JAMA Neurol. 2020;77(6):683–690. 10.1001/jamaneurol.2020.1127 PMC7149362 32275288

[3] Kluge S, Janssens U, Welte T (2020). Empfehlungen zur intensivmedizinischen therapie von patienten mit COVID-19. Med Klin Intensivmed Notfmed.

[4] Wang D, Hu B, Hu C (2020). Clinical characteristics of 138 hospitalized patients with 2019 novel coronavirus-infected pneumonia in Wuhan, China. JAMA.

[5] Windisch W, Weber-Carstens S, Kluge S (2020). Invasive and non-invasive ventilation in patients with COVID-19. Dtsch Arztebl Int.

[6] Westhoff M, Schönhofer B, Neumann P (2015). Nichtinvasive Beatmung als Therapie der Akuten Respiratorischen Insuffizienz. Pneumologie..

[7] Bellani G, Laffey JG, Pham T (2017). Noninvasive ventilation of patients with acute respiratory distress syndrome. Insights from the LUNG SAFE Study. Am J Respir Crit Care Med.

[8] Pfeifer M, Ewig S, Voshaar T (2020). Positionspapier zur praktischen Umsetzung der apparativen Differenzialtherapie der akuten respiratorischen Insuffizienz bei COVID-19. Deutsche Gesellschaft für Pneumologie und Beatmungsmedizin. Pneumologie.

[9] Hui DS, Chow BK, Lo T (2019). Exhaled air dispersion during high-flow nasal cannula therapy versus CPAP via different masks. Eur Respir J.

[10] Guan L, Zhou L, Zhang J (2020). More awareness is needed for severe acute respiratory syndrome coronavirus 2019 transmission through exhaled air during non-invasive respiratory support: experience from China. Eur Respir J.

[11] Landry SA, Barr JJ, MacDonald MI, Subedi D, Mansfield D, Hamilton GS, Edwards BA, Joosten SA (2021). Viable virus aerosol propagation by positive airway pressure (PAP) circuit leak and mitigation with a ventilated patient hood. Eur Respir J.

[12] Associação de Medicina Intensiva Brasileira (AMIB). Orientações sobre o manuseio do paciente com pneumonia e insuficiência respiratória devido a infecção pelo coronavírus (SARS-CoV-2) - Versão n.04/2020*. Available in: https://www.amib.org.br/fileadmin/user_upload/amib/2020/marco/31/0904202_1026_Orientac__o__es_sobre_o_manuseio_do_paciente_com_pneumonia_e_insuficie__ncia_respirato__ria_v4.pdf. Accessed 14 Apr 2021.

[13] Appendini L, Patessio A, Zanaboni S (1994). Physiologic effects of positive end-expiratory pressure and mask pressure support during exacerbations of chronic obstructive pulmonary disease. Am J Respir Crit Care Med.

[14] Marini JJ, Gattinoni L (2020). Management of COVID-19 respiratory distress. JAMA..

[15] Associação de Medicina Intensiva Brasileira (AMIB); Sociedade Brasileira de Pneumologia e Tisiologia (SBPT). Diretrizes Brasileira de Ventilação Mecânica, 2013. Available in: https://www.amib.org.br/fileadmin/user_upload/amib/2018/junho/15/Diretrizes_Brasileiras_de_Ventilacao_Mecanica_2013_AMIB_SBPT_Arquivo_Eletronico_Oficial.pdf. Accessed 13 Apr 2021.

[16] Wang K, Wei Z, Li J (2020). The experience of high-flow nasal cannula in hospitalized patients with 2019 novel coronavirus-infected pneumonia in two hospitals of Chongqing, China. Ann Intensive Care.

[17] Gold JAW, Wong KK, Szablewski CM (2020). Characteristics and clinical outcomes of adult patients hospitalized with COVID-19 - Georgia, March 2020. MMWR..

[18] Antonelli M, Conti G, Moro ML (2001). Predictors of failure of noninvasive positive pressure ventilation in patients with acute hypoxemic respiratory failure: a multi-center study. Intensive Care Med.

[19] Thille AW, Countou D, Fragnoli C (2013). Non-invasive ventilation for acute hypoxemic respiratory failure: Intubation rate and risk factors. Crit Care.

[20] Faul F, Erdfelder E, Lang A-G (2007). G*Power 3: a flexible statistical power analysis program for the social, behavioral, and biomedical sciences. Behav Res Methods.

[21] Martinez JE, Grassi DC, Marques LG (2011). Análise da aplicabilidade de três instrumentos de avaliação de dor em distintas unidades de atendimento: ambulatório, enfermaria e urgência. Rev Bras Reumatol.

[22] Moreno RP, Metnitz PG, Almeida E (2005). SAPS 3-From evaluation of the patient to evaluation of the intensive care unit. Part 2: Development of a prognostic model for hospital mortality at ICU admission. Intensive Care Med.

[23] Chumlea WC, Roche AF, Steinbaugh ML (1985). Estimating stature from knee height for persons 60 to 90 years of age. J Am Geriatr Soc.

[24] Mitchell CO, Lipschitz DA (1982). Arm length measurement as an alternative to height in nutritional assessment of the elderly. J Parenter Enter Nutr.

[25] Roussos C, Koutsoukou A (2003). Respiratory failure. Eur Respir J Suppl.

[26] Holanda MA, Reis RC, Winkeler GFP (2009). Influência das máscaras facial total, facial e nasal nos efeitos adversos agudos durante ventilação não-invasiva. J Bras Pneumol.

[27] Domingues L, Cruz E (2012). Adaptação cultural e contributo para a validação da Escala Patient Global Impression of Change. Ifisionline..

[28] Cohen J (1988). Statistical power analysis for the behavioral sciences.

[29] Chawla R, Dixit SB, Zirpe KG (2020). ISCCM guidelines for the use of non-invasive ventilation in acute respiratory failure in adult ICUs. Indian J Crit Care Med.

[30] Otero DP, Domínguez DV, Fernández LH (2017). Preventing facial pressure ulcers in patients under non-invasive mechanical ventilation: a randomised control trial. Randomized Controlled Trial. J Wound Care.

[31] Fichtner F, Moerer O, Laudi S (2018). Clinical practice guideline: mechanical ventilation and extracorporeal membrane oxygenation in acute respiratory insufficiency. Dtsch Arztebl Int.

[32] Sehgal IS, Kalpakam H, Dhooria S (2019). A randomized controlled trial of noninvasive ventilation with pressure support ventilation and adaptive support ventilation in acute exacerbation of COPD: a feasibility study. COPD..

[33] Nava S, Grassi M, Fanfulla F (2011). Non-invasive ventilation in elderly patients with acute hypercapnic respiratory failure: a randomised controlled trial. Age Ageing.

[34] Hernandez G, Fernandez R, Lopez-Reina P (2010). Non-invasive ventilation reduces intubation in chest trauma-related hypoxemia: a randomized clinical trial. Chest..

[35] Rathi NK, Haque SA, Nates R (2017). Noninvasive positive pressure ventilation vs invasive mechanical ventilation as first-line therapy for acute hypoxemic respiratory failure in cancer patients. J Crit Care.

[36] Nava S, Ferrer M, Esquinas A (2013). Palliative use of non-invasive ventilation in end-of-life patients with solid tumours: a randomised feasibility trial. Lancet Oncol.

[37] Jaber S, Lescot T, Futier E (2016). Effect of noninvasive ventilation on tracheal reintubation among patients with hypoxemic respiratory failure following abdominal surgery: a randomised clinical trial. JAMA..

[38] Patel BK, Wolfe KS, Pohlman AS (2016). Effect of noninvasive ventilation delivered by helmet vs face mask on the rate of endotracheal intubation in patients with acute respiratory distress syndrome: a randomized clinical trial. JAMA..

[39] Reusken CBEM, Broberg EK, Haagmans B (2020). Laboratory readiness and response for novel coronavirus (2019-nCoV) in expert laboratories in 30 EU/EEA countries. Euro Surveill.

